# Multicenter dose de-escalation phase I trial of pressurized intraperitoneal aerosolized chemotherapy (PIPAC) nab-paclitaxel and cisplatin in combination with systemic nab-paclitaxel in recurrent ovarian cancer patients: trial in progress

**DOI:** 10.1515/pp-2025-0036

**Published:** 2025-12-22

**Authors:** Vinita Popat, Paul H. Frankel, Nora H. Ruel, Susan E. Yost, Sue Chang, Edward Wang, Jeannine Villella, Jill Whyte, Richard L. Whelan, Melissa Eng, Raechelle Tinsley, Tim Synold, Ernest Han, Mihae Song, Joshua Cohen, Mustafa Raoof, Thanh Hue Dellinger

**Affiliations:** Department of Surgery, 20220 City of Hope National Medical Center, Duarte, CA, USA; Department of Computation and Quantitative Medicine, City of Hope National Medical Center, Duarte, CA, USA; Department of Medical Oncology and Therapeutics Research, City of Hope National Medical Center, Duarte, CA, USA; Department of Pathology, City of Hope National Medical Center, Duarte, CA, USA; Department of Obstetrics and Gynecology, Northwell Health, New York, NY, USA; Department of Surgery, Northwell Health, New York, NY, USA

**Keywords:** ovarian cancer, phase I trial, PIPAC, nab-paclitaxel, multimodal therapy

## Abstract

**Objectives:**

Ovarian cancer (OC) often presents with peritoneal metastases (PM) which contribute significantly to morbidity and treatment resistance. Pressurized intraperitoneal aerosolized chemotherapy (PIPAC) has emerged as a promising modality for locoregional drug delivery in peritoneal surface malignancies. Historically, combined intraperitoneal (IP) and intravenous (IV) cisplatin and paclitaxel regimens have demonstrated activity in first-line OC treatment. PIPAC nab-paclitaxel and cisplatin with systemic nab-paclitaxel holds promise as a safe and effective therapy, but has not been explored in OC.

**Methods:**

This ongoing, dose de-escalation, single-arm phase I study evaluates triplet bidirectional chemotherapy with a safety lead-in (NCT04329494). The study evaluates the safety and tolerability of a 28-day cycle of Day 1 PIPAC nab-paclitaxel 90 mg/m^2^ and cisplatin 15 mg/m^2^ in combination with Days 8 and 15 IV nab-paclitaxel 100 mg/m^2^ for three cycles in recurrent OC patients with unresectable PM.

**Results:**

Enrollment is ongoing at U.S. academic centers. The primary endpoints are dose-limiting toxicities and adverse events. Secondary endpoints include radiographic (RECIST v1.1), histologic, and surgical response, progression-free and overall survival, and post-operative complications.

**Conclusions:**

This study investigates the safety, feasibility, and preliminary activity of the combination of PIPAC and systemic IV chemotherapy in recurrent OC patients to determine efficacy and safety for a future Phase II trial.

## Article summary

Strengths and limitations of the study:

– First clinical trial to evaluate triplet bidirectional therapy using IV nab-paclitaxel and PIPAC nab-paclitaxel plus cisplatin in ovarian cancer.
– Leverages active drugs in platinum resistant recurrent ovarian cancer with IP therapy, increasing clinical relevance.
– Unlike most published PIPAC ovarian cancer trials to date, this trial combines intravenous and IP therapies to improve extraperitoneal metastatic control, given distant pulmonary, lymphatic, and liver metastatic presentation in most recurrent ovarian cancer patients.
– Incorporates comprehensive response assessment using radiologic, histologic, surgical, and patient-reported outcomes.
– Designed with predefined dose de-escalation and toxicity management criteria to ensure patient safety.
– Efficacy measurement is limited, as expected, from a Phase I trial design, given the limited sample size and single-arm design without a control group.

## Introduction

Ovarian cancer (OC) is the most lethal gynecologic malignancy, with an estimated five-year survival rate of 51 % in the United States [1]. This high mortality rate is largely attributable to the prevalence of peritoneal metastases (PM) at presentation, with over 75 % of patients diagnosed at advanced stages (FIGO III or IV) with widespread carcinomatosis due to nonspecific symptoms [2]. PM are associated with a wide range of complications, including diffuse abdominal pain, malignant ascites, and bowel and urinary tract obstructions, which markedly impaired quality of life and functional status.

Systemic chemotherapy frequently proves less effective in PM due to limited vascularization and the peritoneal-plasma barrier which impedes delivery of chemotherapeutic agents into tumor nodules [3]. These pharmacokinetic limitations result in lower drug concentrations at the tumor site, affecting larger or poorly vascularized nodules, and reducing cytotoxic efficacy.

IP chemotherapy delivers high concentrations of cytotoxic drugs directly into the peritoneal cavity, thereby enhancing locoregional drug exposure and prolonging tumor contact while limiting systemic toxicity. This method takes advantage of the peritoneal-plasma barrier to elevate local drug levels, with agents like cisplatin and paclitaxel demonstrating favorable IP-to-IV area under the curve (AUC) ratios [4]. Especially in ovarian cancer, clinical studies have demonstrated that IP chemotherapy combined with optimal cytoreductive surgery, improves survival patient outcomes, both in normothermic and in hyperthermic conditions [5]. Randomized trials incorporating HIPEC during interval cytoreductive surgery resulted in significantly improved progression-free and overall survival in patients with advanced OC, supporting the use of multimodal management in this disease [6]. While HIPEC has shown benefit in OC patients, limited applicability to the first line setting highlights the need for more tolerable localized therapies that can benefit a recurrent patient population.

**Figure 1:**
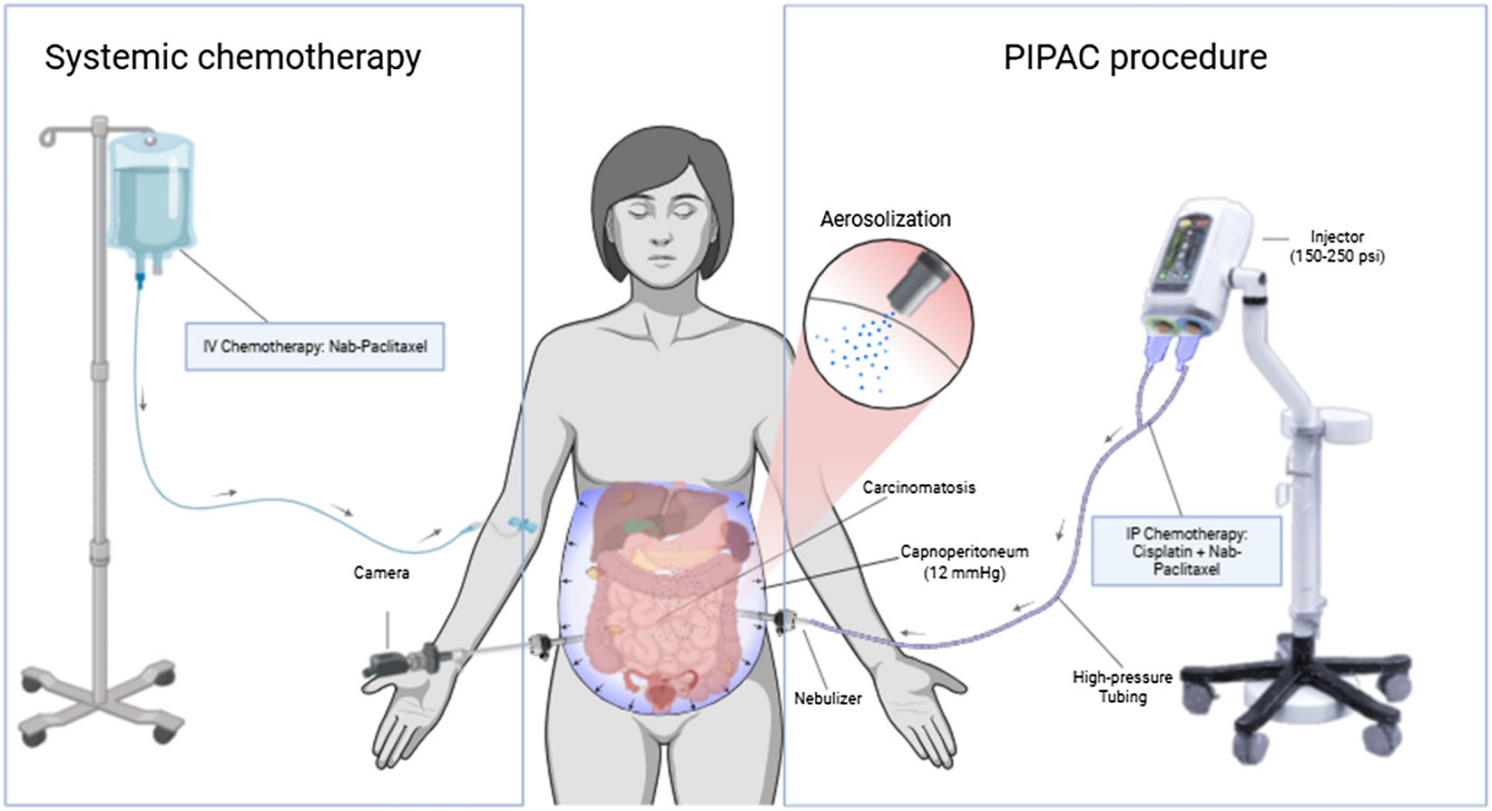
Schema of multimodal drug delivery with PIPAC nab-paclitaxel/cisplatin IP and weekly nab-paclitaxel IV delivered between repeated PIPAC cycles in ovarian cancer patients (created with BioRender.com).

***Pressurized intraperitoneal aerosolized chemotherapy (PIPAC)***: PIPAC is performed laparoscopically using a specialized nebulizer and high-pressure injector to aerosolize chemotherapy into the peritoneal cavity under pressure (Figure 1). PIPAC-delivered chemotherapy enhances tumor tissue penetration, driven by a pressure gradient generated through insufflation and aerosolization, with plasma drug levels approaching equivalent IP dosing. PIPAC combines the pharmacokinetic advantages of IP chemotherapy (high locoregional drug concentration with low systemic exposure) with the improved systemic absorption of an aerosol through a pressure gradient [7], [8], [9], [10]. PIPAC can be administered in multiple treatment cycles, allowing for sustained local-regional treatment, which is particularly valuable in the management of aggressive or recurrent peritoneal cancers. Because of the low drug doses and localized effect, PIPAC has a favorable safety profile with reduced systemic toxicity [10]​.

***Clinical experience with PIPAC in OC***: Since 2011, over 20 retrospective and prospective studies have demonstrated PIPAC’s feasibility, safety, and tolerability in OC patients with PM(11). A systematic review of more than 300 OC patients detailed minimal toxicity and low intraoperative complication rates [11], 12]. This included an 11.5 % incidence of Grade 3–4 postoperative adverse events (CTCAE v5.0), in contrast to >20 % with systemic chemotherapy [13], 14]. Procedure-related mortality was 0.82 %, with intestinal perforation occurring in 2–3 % of procedures [11], 15]. Common adverse events included nausea/vomiting, abdominal pain, and sleep disorders, notably without the significant renal or hepatic toxicities often associated with HIPEC [16], 17].

Early protocols established safe dosing with cisplatin 7.5–10.5 mg/m^2^ and doxorubicin 1.5–2.1 mg/m^2^ delivered at 12 mmHg and 37 °C over 30 min, repeated every 4–6 weeks [18]. While doses up to 30 and 6 mg/m^2^ for cisplatin and doxorubicin, respectively, have been tested without dose-limiting toxicity, the combination of 10.5 and 2.1 mg/m^2^, remains the standard [19], 20].

PIPAC has been most extensively studied in platinum-resistant OC (PROC), where therapeutic options are generally limited. In a phase II trial, Tempfer et al. reported that PIPAC induced a 62 % objective tumor response, 76 % histological regression, and 76 % PCI improvement, despite modest RECIST responses [21]. The phase II PARROT trial also demonstrated a RECIST clinical benefit rate of 82 % in women with PROC, although with modest histologic and laparoscopic scoring response [15].

PIPAC is also being explored in neoadjuvant settings for unresectable stage III–IV OC, where early data suggest potential for downstaging and conversion to surgical candidacy. Some evidence also suggests possible resensitization to platinum agents. The ongoing PIPAC-OVA trial (NCT04811703) is evaluating PIPAC combined with standard systemic chemotherapy to improve cytoreduction rates in platinum-refractory OC patients after initial neoadjuvant chemotherapy [22]. Given PIPAC’s localized delivery and low systemic exposure, it has been investigated in combination with IV chemotherapy, a strategy known as bidirectional therapy, to enhance IP tumor control while treating systemic disease [23].

Despite these promising results, a notable limitation in prior OC PIPAC studies is reliance on cisplatin and doxorubicin. These agents, primarily adopted from palliative HIPEC protocols, do not align with current standard-of-care (SOC) systemic regimens for OC. The other limitation is lack of multimodal therapy with incorporation of intravenous chemotherapy to address extraperitoneal metastases. Most prior OC PIPAC trials excluded patients with extraperitoneal metastases, even though most recurrent OC patients present with distant pulmonary, lymphatic and liver metastases. This gap underscores a critical need for trials that integrate PIPAC with modern systemic therapies to more accurately evaluate its therapeutic value and real-world applicability. Our trial addresses these gaps by incorporating intravenous treatment into multimodal therapy and leveraging clinically active drugs in PROC to improve efficacy of PIPAC.

### Indications for nab-paclitaxel and cisplatin in PIPAC

Nab-paclitaxel: This trial employs nanoparticle albumin-bound paclitaxel (nab-paclitaxel, Abraxane) and cisplatin for PIPAC. Paclitaxel is a cornerstone of first-line systemic chemotherapy SOC treatment modality in the recurrent OC setting [24]. Paclitaxel has activity against peritoneal tumor implants when delivered intraperitoneally [5]. Nab-paclitaxel is a solvent-free, albumin-bound form of paclitaxel with distinct pharmacologic advantages. Nab-paclitaxel avoids cremophor-related toxicities and demonstrates significant single-agent activity even in patients with platinum and taxane-resistant OC [25]. Tumor overexpression of albumin-binding proteins (gp60, SPARC) facilitates deeper drug penetration, a key advantage in treating PM [26], 27]​. Preclinical models confirm superior intratumoral accumulation and cytotoxicity compared to conventional paclitaxel [26]. Pharmacokinetic evaluation of PIPAC nab-paclitaxel demonstrates slow systemic absorption with median peak plasma concentrations at 3–4 h post-procedure and a terminal half-life of ∼8 h [28]. At the maximum tolerated dose (MTD) (140 mg/m^2^), the plasma AUC (0–24 h) was 1905 ng·h/mL–approximately one-third of that observed with equivalent IV dosing, highlighting sustained peritoneal exposure with limited systemic spillover [28]. A Phase I trial using PIPAC with nab-paclitaxel for the treatment of PM from multiple primary cancer types, including ovarian, demonstrated gradual intratumoral accumulation of chemotherapy over multiple PIPAC cycles with limited systemic absorption [28]. Notably, 35 % of patients had objective tumor response and 35 % had disease stabilization, supporting its activity against PM [28]. However, systemic chemotherapy was administered per investigator discretion, limiting the ability to assess feasibility and safety specifically in recurrent OC. This study laid the groundwork for the Nab-PIPAC Phase Ib trial (NCT04000906), which is currently evaluating the safety, tolerability, and pharmacokinetics of escalating doses of IP nab-paclitaxel (7.5–70 mg/m^2^) in combination with cisplatin via PIPAC in gastric, pancreatic or ovarian cancer patients; no systemic (bidirectional) therapy is included in this study [29].

Cisplatin: Cisplatin forms the backbone of both HIPEC and normothermic IP trials in OC, as well as in many PIPAC trials. PIPAC-delivered cisplatin promises effectiveness in platinum resistance, producing meaningful objective responses and durable disease stabilization in heavily pre-treated, platinum-resistant patients while maintaining an excellent safety profile [19], 21], 30]. Pharmacokinetic analyses show rapid systemic absorption and effective tumor penetration, with platinum tissue concentrations increasing over threefold post-PIPAC and plasma drug exposure approaching levels seen with IV administration [4]. Elevated intra-abdominal pressure further boosts the unbound fraction of cisplatin by ∼20 %, driving peak plasma exposure to just below that of an equivalent IV dose, a key determinant of antitumor efficacy [31], 32]. Collectively, these clinical and pharmacologic data justify the use of PIPAC cisplatin as a potent, safe, and mechanistically sound strategy for treating PM in OC.

Combination of nab-paclitaxel with cisplatin: IP and systemic taxane and cisplatin combinations are effective in OC, as demonstrated by multiple intraperitoneal OC trials, including the Armstrong trial. This randomized phase 3 trial compared IV cisplatin and paclitaxel with IV paclitaxel plus IP cisplatin and paclitaxel in optimally debulked stage III OC patients, and reported significantly improved progression-free and overall survival in the IV/IP cohort [33]. These findings underscore the IP/IV platinum/taxane combination as an active OC treatment modality.

In this study, we will determine if standard OC chemotherapy – when used systemically and intraperitoneally in a coordinated, protocolized manner – can deliver greater disease control in recurrent OC patients undergoing PIPAC. Our trial is the first to evaluate guideline-based nab-paclitaxel in both intraperitoneal and extraperitoneal compartments for patients with peritoneal-dominant OC. PIPAC with nab-paclitaxel has not been extensively studied in OC, and its role in combination with systemic chemotherapy is yet to be elucidated. We hypothesize that PIPAC nab-paclitaxel and cisplatin in combination with systemic nab-pacliltaxel will be safe and tolerable, with the goal to identify the MTD and recommended Phase 2 dose (RP2D).

## Methods and analyses

Trial design: This ongoing, multicenter Phase 1 trial assesses the safety and feasibility of combining PIPAC nab-paclitaxel and cisplatin with systemic weekly nab-paclitaxel in recurrent OC patients who present with PM and are not candidates for cytoreductive surgery at City of Hope, U.S.A. and affiliate sites. Current affiliate sites include Northwell Health, NY. Reporting checklist for the protocol of a clinical trial was provided using SPIRIT reporting guidelines [34].

Study population and recruitment: This study is intended for patients with platinum resistant or refractory recurrent OC with PM who are ≥18 years of age, Eastern Cooperative Oncology Group (ECOG) performance status (PS) ≤2, and no contraindications to laparoscopy. Patients must have progressed on at least one line of an evidence-based chemotherapy regimen prior to enrolling on the study. Exclusion criteria include bowel obstruction, prior severe reaction, or hypersensitivity to platinum-based or taxane-based compounds, chemotherapy within the last 4 weeks (6 weeks for bevacizumab therapy), life expectancy <6 months, severe medical comorbidities or laboratory abnormalities, and major systemic infection (Table 1). Patients must be at least 6 months post first-line SOC chemotherapy with no bowel obstruction.

**Table 1:** Inclusion and exclusion criteria.

Inclusion criteria	Exclusion criteria
– Documented informed consent – ≥18 years old – Histologically confirmed ovarian, fallopian tube, or primary peritoneal carcinoma – Recurrent disease with peritoneal metastases (with or without limited extra-peritoneal disease) – Visible peritoneal disease on imaging or laparoscopy – Prior platinum-based chemotherapy (≥1 line) with no curative surgical options remaining. – ECOG performance status ≤2. – Adequate hematologic and end-organ function (renal, hepatic, cardiac) to undergo chemotherapy. – Able to undergo laparoscopy (no prohibitive adhesions or surgical risk)	– Extensive extra-peritoneal metastases – Bowel obstruction – Exclusive total parenteral nutrition – Simultaneous tumor debulking with gastrointestinal resection – Severe allergy or hypersensitivity to platinum agents or taxanes – Pre-existing grade ≥2 peripheral neuropathy or unresolved toxicities from prior therapy that would be exacerbated by study treatment – Uncontrolled serious medical conditions that would pose unacceptable risk for anesthesia or study treatment. – Pregnancy or breastfeeding – Patients unable to comply with study procedures or follow-up – Life expectancy of less than 6 months

PIPAC procedure and systemic chemotherapy: A detailed description of technical considerations (including occupational safety) for PIPAC has been previously described [35]. During laparoscopy, 12 mm and 5 mm balloon ports are placed to ensure adequate seal and prevent leakage of pneumoperitoneum. Ascites is removed, PCI determined, and pre-treatment peritoneal biopsies taken in all accessible quadrants. No lysis of adhesions or tumor resection is performed due to increased morbidity. Cisplatin 15 mg/m^2^ dissolved in 150 mL of 0.9 % NaCl and nab-paclitaxel 90 mg/m^2^ dissolved in 150 mL of 0.9 % NaCl is used for the PIPAC procedure. Prior to administering nab-paclitaxel, the reconstituted suspension is visually inspected and if proteinaceous strands are observed, the suspension is discarded. A high-pressure injector and nebulizer is employed for drug administration, operating at an average of 150 psi and 30 mL/min. Following aerosolization, the agents are allowed to precipitate within the peritoneal cavity for 30 min at room temperature, after which any remaining aerosol will be evacuated.

**Figure 2:**
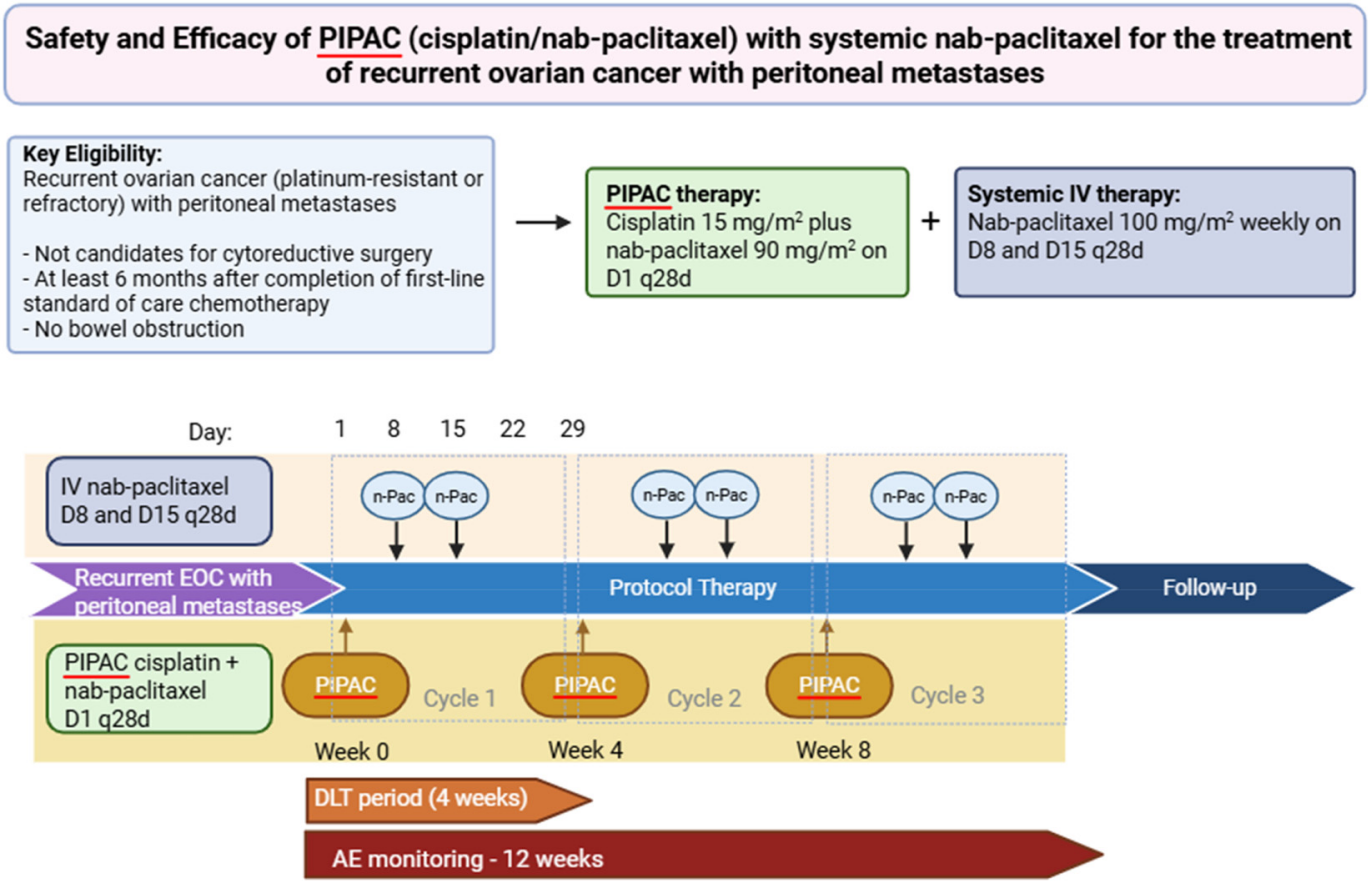
Study schema for PIPAC cisplatin/nab-paclitaxel followed by systemic nab-paclitaxel in recurrent ovarian cancer patients with peritoneal metastases. PIPAC cisplatin (15 mg/m^2^) and nab-paclitaxel (90 mg/m^2^) are administered on D1 q28d, followed by systemic IV nab-paclitaxel (100 mg/m^2^) weekly D8 and D15 every 28 days (created with BioRender.com).

IV nab-paclitaxel 100 mg/m^2^ is administered starting one week after the first PIPAC on day 8 and day 15 of every 28-day cycle (Figure 2). Three cycles of PIPAC with cisplatin and nab-paclitaxel with concurrent IV nab-paclitaxel is administered. For patients with demonstrated clinical benefit (e.g. partial response by CA-125 or RECIST measurement) from PIPAC + IV nab-paclitaxel, up to three additional PIPAC cycles with continuation on IV chemotherapy is allowed (up to 6 cycles total). Weekly IV systemic chemotherapy may be continued after 15 weeks, off trial, without PIPAC in standard fashion per treating physician’s discretion. The safety (dose limiting toxicity) evaluation period is 4-weeks after initial treatment, and a delay in PIPAC administration over 21 days is considered a dose limiting toxicity (DLT), with plans for dose de-escalation as needed.

Rationale for IP cisplatin dosage: A Phase I trial from Italy demonstrated an MTD of 30 mg/m^2^ for cisplatin in combination with doxorubicin 6 mg/m^2^ for PIPAC delivery for a small cohort of gastrointestinal and OC patients [20]. However, this study did not report MTD in combination with systemic chemotherapy. A recent phase I trial in India with a larger OC cohort demonstrated safety and efficacy of PIPAC using cisplatin 15 mg/m^2^ in combination with doxorubicin [30]. Based on the experience of these prior studies, cisplatin 15 mg/m^2^ was deemed appropriate for this PIPAC trial with plans for dose de-escalation as needed.

Rationale for IP nab-paclitaxel dosage: In a previous study conducted at our institution, IP nab-paclitaxel in non-aerosolized (non-PIPAC) delivery was evaluated in solid tumors with excellent tolerability and promising activity, with a reported MTD of 140 mg/m^2^ [5]. Following this experience, PIPAC with nab-paclitaxel was evaluated in a first-in-human phase I trial by W. Ceelen et al. [28]. The recommended dose of PIPAC with nab-paclitaxel was 140 mg/m^2^, however several patients had incisional wound complications [28]. Additionally, although IV chemotherapy was included in this study, the choice of agent was left to the investigator’s discretion. This resulted in a heterogeneous treatment population, making it difficult to isolate the contribution of systemic therapy versus PIPAC to observed outcomes. Given the risk of wound-related complications and the potential for increased systemic exposure from combined IV and IP nab-paclitaxel, both of which are associated with marrow toxicity, we elected to begin with a reduced PIPAC nab-paclitaxel dose of 90 mg/m^2^.

Rationale for IV nab-paclitaxel dosage: Weekly IV nab-paclitaxel is SOC therapy for recurrent OC, and the systemic backbone for this IV/IP study. In OC, IV nab-paclitaxel is frequently administered at a dose of 100 mg/m^2^, which is the same dose we plan to utilize in our study [25]. While typically three weekly doses of IV nab-paclitaxel are delivered, we chose to administer two weekly IV doses per cycle, as the initial PIPAC nab-paclitaxel dose represents the first of the three weekly doses of nab-paclitaxel.

Dose de-escalation: This study employs a tiered dose de-escalation strategy with three predefined dose levels for IV nab-paclitaxel, PIPAC-administered cisplatin, and PIPAC-administered nab-paclitaxel (Table 2). The initial dose level (DL1) consists of IV nab-paclitaxel at 100 mg/m^2^, PIPAC cisplatin at 15 mg/m^2^, and PIPAC nab-paclitaxel at 90 mg/m^2^. If toxicities emerge, de-escalation proceeds stepwise to DL-1 and DL-2. DLTs include surgical complications of Clavien–Dindo grade IIIB or higher, treatment delays exceeding 21 days due to PIPAC-related toxicity, or severe hematologic/non-hematologic adverse events (CTCAE v5.0). Dose modifications are made according to prespecified criteria (Table 3), including ANC thresholds, platelet count, neuropathy, gastrointestinal symptoms, and cutaneous or pulmonary events. Hematologic toxicity may prompt dose delays or permanent dose reductions in both IV and PIPAC nab-paclitaxel; non-hematologic toxicity ≥Grade 3 requires holding the drug until resolution and resuming at a lower dose. As the systemic nab-paclitaxel is administered as a SOC systemic treatment, all other dose modifications for IV nab-paclitaxel will occur per physician discretion. G-CSF support is allowed at physician discretion. Once a dose level is reduced, re-escalation is not allowed. This framework ensures patient safety while enabling continuous delivery of triplet bidirectional therapy.

**Table 2:** Dose de-escalation strategy.

Dose level	IV nab-paclitaxel, mg/m^2^	PIPAC cisplatin, mg/m^2^	PIPAC nab-paclitaxel, mg/m^2^
DL1 (standard)	100	15	90
DL-1	80	10	70
DL-2	60	7.5	50

**Table 3:** Dose modification criteria. Dose modifications for IV nab-paclitaxel are at physician discretion except in the setting of neutropenia.

Toxicity type	Grade	Modification
Hematologic	ANC <1,000/mm2 or platelets <75,000/μL	Delay treatment by 1-week intervals until recovery
	Second occurrence	Delay until recovery, then reduce both IV (only for neutropenia) and PIPAC nab-paclitaxel by one dose level
Non-hematologic	Grade 3 or 4 (excluding alopecia, manageable nausea)	Hold PIPAC nab-paclitaxel until <grade 1, then resume at next lower dose level
Sensory neuropathy	Persistent grade 2	Decrease PIPAC nab-paclitaxel by one dose level
	Grade 3	Hold until <grade 2, then resume at one dose level lower
	Grade 4	Hold until <grade 2, then resume at two dose levels lower
Cutaneous or GI toxicity	Grade 2 or 3	Reduce dose of the most likely causative agent
	Persistent despite reduction	Discontinue treatment
	Grade 4	Discontinue treatment

Trial duration: Each subject will receive up to 3 PIPAC treatment cycles (4–6 weeks apart). Follow-up for progression-free survival (PFS) will continue every 3 months through an expected time period of 1 year, and patients who are still progression-free at 1 year will continue to be followed for progression. The expected duration of the trial is approximately 2.5 years.

Statistics: The planned sample size for this study arm includes approximately 6 patients in the safety lead-in (assuming DL1 is well-tolerated). The safety lead-in accrual rules follow the IQ 3 + 3 dose de-escalation design to define the MTD or RP2D [36]. Based on operating characteristics across multiple toxicity scenarios, this cohort is expected to enroll 5–15 patients during dose-finding. This IQ 3 + 3 design is used to reduce study duration when compared to the traditional 3 + 3 by taking into account the patient queue, specifically addressing screen failures, unevaluable patients (which are not infrequent in IP studies) and the accrual rate while maintaining the level of patient risk consistent with the traditional 3 + 3 design. It also allows for more rapid integration of evaluable patients from the dose-finding portion into the dose expansion phase, where we estimate approximately 20 patients will be accrued (initially designed as formal 2-stage Phase 2 design with a total of 25 patients at the RP2D), but it is currently expected to close earlier due to funding/support (e.g. limitation on the number of PIPAC nebulizer devices available).

Median PFS will be estimated using the Kaplan-Meier method and evaluated for each dose level as well as the entire cohort. Exploratory pharmacokinetic (PK) and pharmacodynamic (PD) analyses will be performed to identify potential correlations with clinical activity. Given the exploratory nature of these analyses, results will be interpreted with appropriate caution, including acknowledgment of multiple comparisons. Safety and toxicity will be summarized descriptively by severity and attribution. This statistical framework supports efficient dose selection, early efficacy assessment, and hypothesis generation while maintaining patient safety.

## Results

Recruitment: Participants are being recruited at multiple sites throughout the United States including City of Hope National Medical Center and Northwell Health. All investigators received PIPAC certification by the International Society for the Study of Pleura and Peritoneum (ISSPP). Both sites were accruing OC patients for PIPAC cisplatin and doxorubicin administration since 2020 and have implemented the nab-paclitaxel PIPAC protocol as a fourth arm of the US PIPAC trial (NCT04329494).

Primary endpoints: The primary endpoint of this study is safety, evaluated through predefined rules for toxicity (dose limiting toxicity) based on the IQ 3 + 3 design. Each patient cohort will be monitored for adverse events within a four-week observation window following each PIPAC cycle. Toxicities will be summarized by PIPAC dose level (and systemic therapy dose for applicable cycles), categorized by organ system or laboratory parameter, graded by severity (per CTCAE v5.0), and assessed for attribution, onset timing, duration, association with study treatment, and reversibility. Postoperative surgical complications will be monitored for 28 days following each PIPAC procedure and classified using the Clavien–Dindo system [37]. These data will inform tolerability and guide decisions on dose modification or discontinuation under the protocol’s safety management plan.

Secondary endpoints: Secondary endpoints will evaluate preliminary efficacy, procedure-related outcomes, and patient-centered measures. Efficacy will be assessed using RECIST v1.1 on CT scans performed at baseline, week 10, six weeks post-treatment, and at week 18, and defined as the percentage of patients who achieved CR, PR, or SD; histologic tumor response will be evaluated using the peritoneal regression grading score (PRGS) based on pre- and post-PIPAC peritoneal biopsies at each cycle; and changes in peritoneal tumor burden will be measured using the peritoneal cancer index (PCI) at each laparoscopy. Additional endpoints include post-operative complications classified by Clavien–Dindo criteria, PIPAC technical failure rate, and progression-free and overall survival.

## Discussion

This trial introduces a novel multi-modal therapeutic strategy for recurrent OC patients with PM, integrating IV nab-paclitaxel with PIPAC-delivered cisplatin and nab-paclitaxel. This is, to our knowledge, the first clinical protocol to evaluate this specific triplet multi-modal regimen in ovarian cancer, combining systemic and regional delivery of chemotherapy in a structured dose de-escalation framework. The rationale is grounded in both the pharmacologic strengths of nab-paclitaxel – enhanced tumor penetration via albumin-mediated uptake and avoidance of solvent-related toxicity – and the favorable locoregional pharmacokinetics of PIPAC.

Previous PIPAC trials have evaluated heterogeneous patient populations encompassing various gastrointestinal and ovarian cancers, with IV chemotherapy administered per investigator discretion [28]. This heterogeneity complicates the interpretation of feasibility and safety outcomes specific to OC. Furthermore, aside from the two nab-paclitaxel PIPAC trials mentioned above, most PIPAC studies in OC have employed cisplatin/doxorubicin combinations, which are not standard IP chemotherapy regimens for this disease, and have demonstrated modest responses in published reports. Our study distinguishes itself by utilizing weekly IV nab-paclitaxel, an SOC treatment in recurrent OC, thereby aligning IP therapy more closely with established OC treatment protocols [29]. In this context, PIPAC may serve a similar role to bevacizumab-containing regimens for peritoneal disease control. While bevacizumab is a frequently used drug in OC, some patients with advanced OC are ineligible for bevacizumab due to contraindications such as venous thromboembolism, recurrent bowel obstruction, uncontrolled hypertension, or proteinuria. Moreover, bevacizumab is associated with substantial toxicity, with all-grade adverse events reported in up to 57 % of treated patients [38]. For this patient subset, PIPAC thus presents a compelling alternative, characterized by its localized drug delivery, reduced systemic exposure, and a generally favorable safety profile.

The design of our trial has been directly shaped by critical lessons gleaned from prior studies. For example, the study by Ceelen et al. reported a 40 % incidence of wound complications, underscoring the need for meticulous surgical technique and vigilant postoperative management [28]. Additionally, weekly administration of nab-paclitaxel on a 28-day cycle presents challenges, including cumulative marrow suppression and risk of pancytopenia. These concerns – combined with the potential for impaired wound healing and systemic absorption of intraperitoneally delivered nab-paclitaxel – have prompted us to initiate treatment with a lower starting dose to optimize safety while maintaining feasibility.

This study targets a population with significant unmet need: patients with platinum-resistant recurrent OC, where systemic options are often exhausted. These patients present with additional extraperitoneal disease, which are not addressed by IP therapy such as PIPAC alone. The protocol is designed with predefined dose levels and toxicity management criteria to ensure safety while maintaining adequate IP drug exposure. Primary endpoints focus on safety and feasibility, which are essential for establishing the regimen’s clinical viability. In addition to safety, the protocol incorporates a broad set of exploratory efficacy and patient-centered endpoints. These include histologic (PRGS), radiographic (RECIST), and surgical (PCI) assessments, as well as quality-of-life metrics and physical activity monitoring. Such multidimensional outcome measures will provide insight into not only biological response, but also the functional impact of therapy.

Should this approach prove successful, it could establish a crucial foundation for integrating multi-modal chemotherapy into future treatment paradigms for peritoneal-dominant recurrent OC, particularly in scenarios where conventional systemic options offer limited benefit.
